# Morphological Transition in Diamond Thin-Films Induced by Boron in a Microwave Plasma Deposition Process

**DOI:** 10.3390/ma10111305

**Published:** 2017-11-14

**Authors:** Paul A. Baker, David R. Goodloe, Yogesh K. Vohra

**Affiliations:** 1 Department of Physics, University of Alabama at Birmingham, Birmingham, AL 35294, USA; pabaker@uab.edu; 2 Department of Chemistry and Physics, Birmingham Southern College, Birmingham, AL 35254, USA; drgoodlo@icloud.com

**Keywords:** microwave plasma deposition, nanostructured diamond, optical emission spectroscopy, *x*-ray diffraction, microcrystalline diamond

## Abstract

The purpose of this study is to understand the basic mechanisms responsible for the synthesis of nanostructured diamond films in a microwave plasma chemical vapor deposition (MPCVD) process and to identify plasma chemistry suitable for controlling the morphology and electrical properties of deposited films. The nanostructured diamond films were synthesized by MPCVD on Ti-6Al-4V alloy substrates using H_2_/CH_4_/N_2_ precursor gases and the plasma chemistry was monitored by the optical emission spectroscopy (OES). The synthesized thin-films were characterized by *x*-ray diffraction and scanning electron microscopy. The addition of B_2_H_6_ to the feedgas during MPCVD of diamond thin-films changes the crystal grain size from nanometer to micron scale. Nanostructured diamond films grown with H_2_/CH_4_/N_2_ gases demonstrate a broad (111) Bragg *x*-ray diffraction peak (Full-Width at Half-Maximum (FWHM) = 0.93° 2θ), indicating a small grain size, whereas scans show a definite sharpening of the diamond (111) peak (FWHM = 0.30° 2θ) with the addition of boron. OES showed a decrease in CN (carbon–nitrogen) radical in the plasma with B_2_H_6_ addition to the gas mixture. Our study indicates that CN radical plays a critical role in the synthesis of nanostructured diamond films and suppression of CN radical by boron-addition in the plasma causes a morphological transition to microcrystalline diamond.

## 1. Introduction

The nanostructured diamond deposition on a variety of metallic and non-metallic substrates is important for applications in improving materials performance under extreme conditions of pressure, temperature, mechanical wear, radiation, and corrosion [1]. The nanostructured diamond deposition has been achieved under a variety of plasma conditions including the use of hydrogen/methane/nitrogen gas phase mixtures, however, the detailed mechanism of synthesis and morphological control is not well understood [2]. In parallel, addition of nitrogen and boron impurities during chemical vapor deposition (CVD) of diamond films has been extensively studied to modify the electrical properties of diamonds. The focus mainly has been on the addition of nitrogen in diamonds to make them N-type and the creation of nitrogen-vacancy centers for quantum computing applications. The addition of boron in diamonds has been studied to make it P-type semiconductor. These additions create sites in the lattice with either an extra electron (the case of the nitrogen atoms) or have one less electron than the surrounding carbon atoms (as in the case of the boron atoms). The acceptor level induced by boron in diamonds is about 0.37 eV above the valence band [3] which combined with its excellent thermal conductivity makes it a very good candidate as a material for semiconducting devices. The addition of boron at relatively low concentrations (~10^7^ atoms/cm^3^) has been shown to change the resistivity of diamond films from 10^8^ Ω-cm to 10^5^ Ω-cm [4], and at boron concentrations of ~4.5 × 10^20^ atoms/cm^3^ the diamond has a resistivity of 1.43 × 10^−2^ Ω-cm [5]. This behavior has led researchers to look for superconducting properties in boron-doped diamonds (BDD) and it has been demonstrated that they has Tc~4 K [6]. 

The incorporation of boron into the diamond film has also been studied but not conclusively determined. It can incorporate substitutionally in the diamond lattice creating an acceptor electron site [7], but also the atoms can cluster at grain boundaries [8]. The level of boron doping can affect where the boron atoms end up in the film as well. At low boron doping levels the boron typically incorporates substitutionally whereas at higher doping levels it begins to cluster in the grain boundaries. The influence of substrate temperature and other conditions during deposition such as plasma temperature and chamber pressure (which would change the composition of the ionized particles in the plasma) have not been fully studied. 

Nitrogen addition has also been shown to decrease the resistance of diamond films near to that of heavily BDD [9]. This has been shown to be a result of grain boundary conduction resulting from an increase in the grain boundary volume, and the incorporation of nitrogen in the film remained low (<1 at %) even with extremely high doping concentrations (N_2_ = 20% of gas flow) [10].

The grain size of the diamond crystals in the film can be on the order of microns or of nanometers depending on the gas-phase chemistry and other deposition conditions. If the application for the film requires reducing mechanical wear then a smaller grain size is more desirable. It has been found that the addition of nitrogen has the added effect of shrinking the crystal size using roughly the same deposition conditions as would normally produce micron sized crystals [11]. The nitrogen appears to affect the crystal growth in a catalytic way in that nitrogen is present in the plasma but gets incorporated in a very low concentration. Thus, this makes the addition of nitrogen an easy method for producing a smooth film (Root Means Squared (RMS) surface roughness ≈ 14 nm).

Combining these two techniques in order to produce P-type, low surface roughness, diamond thin-films was the next logical step. Applications for this type of film on Ti-6Al-4V (a widely used alloy in the aerospace and biomedical industries) are for the protection of electrical contacts in extreme environments by preventing corrosion and providing wear resistance. The motivation of this study is to understand the basic mechanism responsible for the synthesis of nanostructured diamond films and to utilize nitrogen and boron related species in the plasma to control the morphology and electrical properties of the films.

## 2. Results

The purpose of the experiments was to demonstrate clearly the differences between three different types of diamond films: microcrystalline (labeled MCD, H_2_/CH_4_ precursors), nitrogen induced nanostructured (labeled NSD, H_2_/CH_4_/N_2_ precursors), and simultaneously added boron and nitrogen (labeled BND, H_2_/CH_4_/N_2_/B_2_H_6_ precursors). A fourth film containing only boron dopant (labeled BDD, H_2_/CH_4_/B_2_H_6_ precursors) was grown for comparison purposes, to calculate the boron concentration. The grown films were characterized by thin-film *x*-ray diffraction and scanning electron microscopy and plasma diagnostics was performed by optical emission spectroscopy. Each of the different diamond films exhibited clear distinctions from the rest in terms of surface morphology or crystal structure.

**Figure 1:**
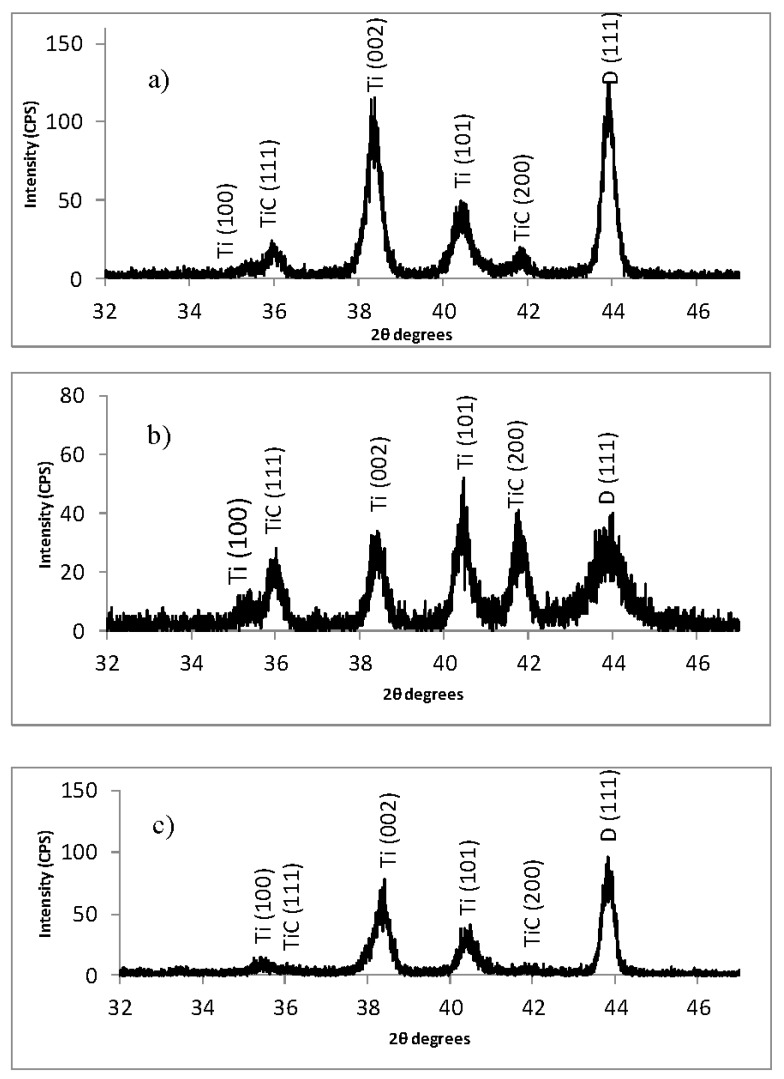
Thin-film *x*-ray diffraction patterns recorded with Cu K_α_ radiation for diamond films on Ti-6Al-4V substrates. The diffraction peaks are identified for hcp Ti-alloy, cubic TiC, and cubic diamond (D) phases. (**a**) Microcrystalline diamond (MCD); (**b**) nanostructured diamond (NSD); (**c**) diamond deposited with nitrogen and boron (BND) exhibiting microcrystalline morphology.

Figure 1 shows glancing angle *x*-ray diffraction patterns of MCD (Microcrystalline diamond), NSD (nanostructured diamond), and BND (Boron–Nitrogen Diamond) thin-films grown on Ti-6Al-4V alloy substrate. Three different crystallographic phases can be identified in Figure 1, hexagonal closed packed phase of Ti-6Al-4V alloy as indicated by (100), (002) and (102) diffraction peaks, cubic titanium carbide (TiC) phase indicated by (111) and (200) diffraction peaks, and cubic diamond phase as indicated by a strong D(111) peak.

**Figure 2:**
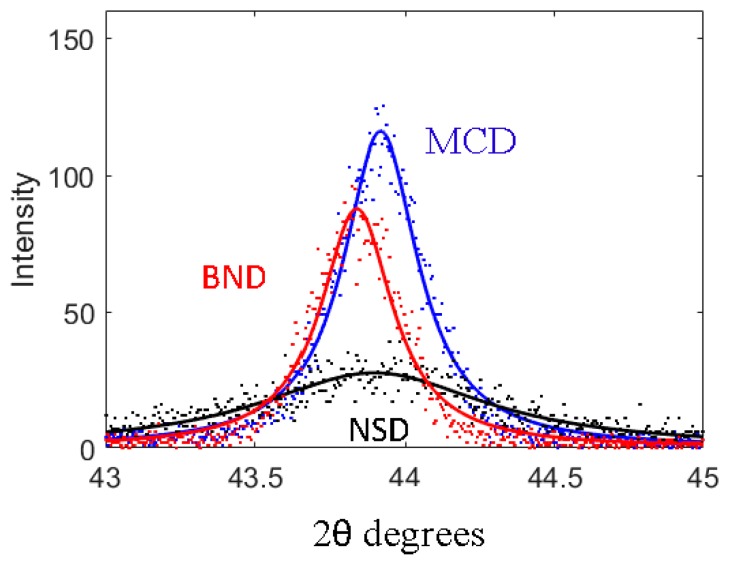
Observed and fitted profile for (111) Bragg diffraction peak for cubic diamond for MCD, NSD, and BND films.

The D(111) for NSD diamond shows a very broad peak typical of nanostructured diamond while the D(111) peak for both MCD and BND are sharp and show a very similar profile. It is well known that *x*-ray diffraction peak broadening has three main contributions: instrumental broadening, crystal size broadening, and strain broadening. The instrumental broadening should be the same for all scans because they were all performed using the same settings on the same instrument. The strain broadening should also be similar for all scans because the growth conditions (temperature, power, and pressure) for the films were all the same and therefore the residual stress in the films is similar for all samples. This leaves the contribution from crystal size broadening. The broadening is further illustrated in Figure 2 where a close-up of the D(111) peak is presented and the contrast in the peak-width between the NSD and MCD/BND is very clear. The fitted profile in Figure 2 shows that broad (111) Bragg *x*-ray diffraction peak for NSD has a Full Width at Half Maximum (FWHM = 0.93° 2θ), indicating a small grain size, whereas scans show a definite sharpening of the diamond (111) peak for BND (FWHM = 0.30° 2θ) with the addition of boron. The measured values for FWHM of the D(111) peaks and lattice parameters are shown in Table 1.

**Figure 3:**
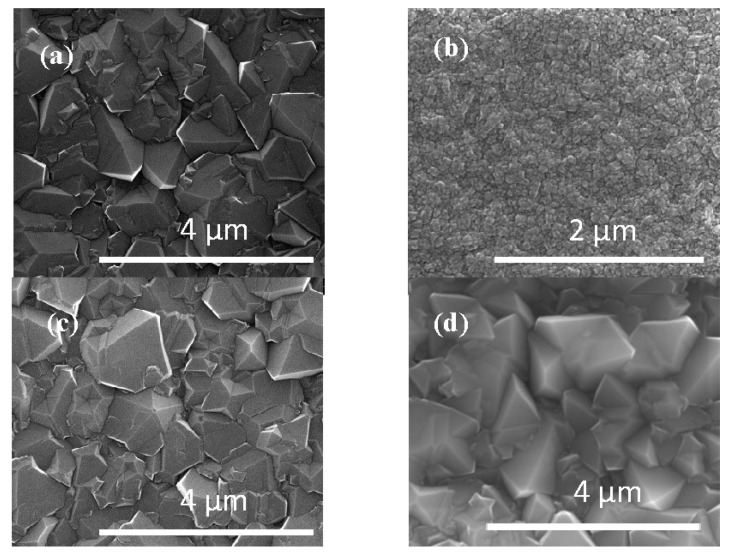
Scanning Electron Microscopy (SEM) of (**a**) Microcrystalline Diamond (MCD); (**b**) Nanostructured Diamond (NSD) (**c**) Boron–Nitrogen Diamond (BND), and (**d**) Boron-Doped Diamond (BDD).

It is important to note that even though D(111) peaks for both MCD and BND have similar widths the D(111) the peak for BND is shifted to lower 2θ values in comparison to MCD in Figure 2 due to a slight increase in diamond lattice parameter due to boron doping. This shift in lattice parameter can also be used to calculate the doping level in the lattice using the equation outlined by Brunet et al. where Δ*a*/*a*_0_ = −5.6 × 10^−4^ + 2.74 × 10^−24^ [B] [12]. Here *Δa* =*a − a*_0_, *a* and *a*_0_ are the lattice parameters for the BND and NSD diamond films, respectively, and [B] is the boron concentration. Based on the lattice parameter shift, the doping level in the BND sample was calculated to be approximately 8 × 10^20^ atoms/cm^3^. Based on this doping level, we anticipate the electrical resistivity of our BND films to be 1.43 × 10^−2^ Ω-cm [5]. For comparison the BDD sample was calculated to have a doping level of 8.5 × 10^20^ atoms/cm^3^ using the lattice parameters for BDD and MCD. The boron-doping levels are very similar BND and BDD samples. This level of doping is consistent with other researchers’ findings for small (ppm level) boron gas concentrations [13]. Figure 3 shows the Scanning Electron Microscopy (SEM) results for MCD, NSD, BND, and BDD thin films grown on Ti-6Al-4V alloy substrates. The measured average grain size for MCD and BND is 800 nm while the NSD film has an average grain size of 60 nm. The BBD film had a grain size of approximately 1 μm.

**Figure 4:**
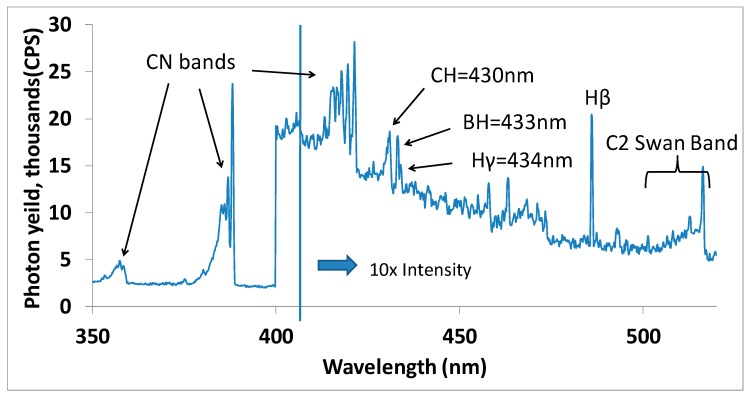
Optical emission spectrum from a H_2_/CH_4_/N_2_/B_2_H_6_ plasma during a microwave plasma deposition experiment. The microwave power in 1.2 kW and the chamber pressure is 38 torr.

A detailed plasma diagnostics study using optical emission spectroscopy (OES) was undertaken to identify the chemical species responsible for the morphological transition in diamond films. OES spectra were collected for plasma containing 500 Standard Cubic Centimeters per Minute (SCCM) of hydrogen, 88 SCCM of methane, 8.8 SCCM of nitrogen and with diluted diborane flow rate varying from 0 to 3 SCCM. Figure 4 shows OES spectra recorded between 350 nm to 520 nm identifying main plasma emission peaks corresponding to hydrogen (H_β_ and Hγ), carbon dimer (C_2_), carbon–nitrogen (CN), carbon–hydrogen (CH) and boron–hydrogen (BH) species.

**Figure 5:**
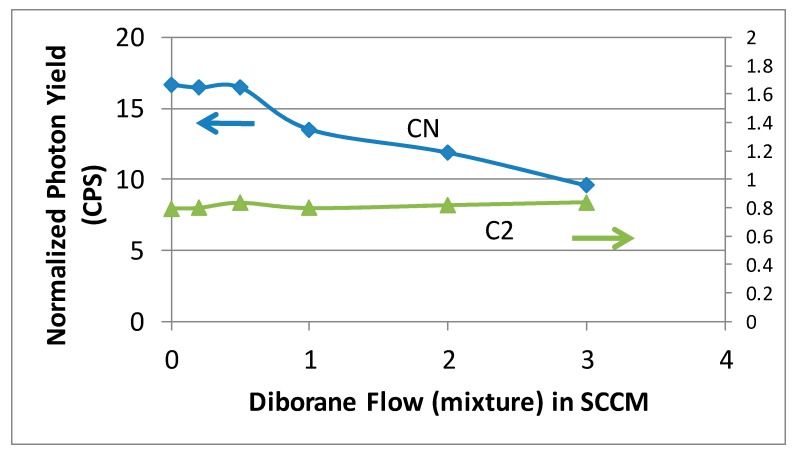
The emission intensity for CN (carbon–nitrogen) and C_2_ radical as a function of diluted diborane mixture (10% B_2_H_6_ in H_2_) flow rate in a H_2_/CH_4_/N_2_ plasma. The emission intensity has been normalized to the hydrogen H_β_ intensity to account for any changes in optical transmission during the course of measurements.

The data in Figure 4 were employed to obtain integrated intensities for CN (388.1 nm) and C_2_ (516.3 nm) peaks as a function of diluted diborane mixture (10% B_2_H_6_ in H_2_) flow rate varying from 0 to 3 SCCM. The intensities were normalized to H_β_ intensity to account for any variation in optical transmittance during the time duration of measurements and are plotted in Figure 5.

It is clearly seen in Figure 5 that the measured intensities of CN species show a marked decrease with increasing diborane (B_2_H_6_) flow rate while the carbon-dimer (C_2_) concentration remains unchanged with increasing B_2_H_6_ flow rate. The CN radical is believed to contribute to nanostructured diamond formation by restricting growth of existing crystals by rapid hydrogen abstraction and promoting re-nucleation of diamond crystallites [14]. This study has clearly identified the role of CN species in the synthesis of nanostructured diamond and suppression of CN species by addition of boron to the plasma.

## 3. Discussion

There has been a long-term interest in the mechanism behind the formation of nanostructured diamond films on a variety of metallic and non-metallic substrates. Various models based on carbon-dimer (C_2_) and carbon–nitrogen (CN) species have been proposed. Our systematic experimental studies with very low level of diborane (170 ppm level) addition to a hydrogen/methane/nitrogen plasma have identified a morphological transition in grain size from nanometer to micron scale in diamond films deposited on Ti-6Al-4V substrates. Optical emission spectroscopy has revealed a systematic decrease in CN radical with an increase in diborane concentration and thus identified CN as a primary radical promoting nano-crystallinity in films grown with hydrogen/methane/nitrogen chemistry. The drop in CN radical is attributed to a rise in the presence of BN radical on addition of diborane to the plasma. This is currently being investigated further using gas phase thermodynamic equilibrium calculations involving H_2_/CH_4_/N_2_/ B_2_H_6_ mixtures.

## 4. Materials and Methods

All of the samples in this study were grown in a 6 kW microwave plasma chemical vapor deposition (MPCVD) system made by Wavemat Inc. (Plymouth, MI, USA). The diamond films were grown on polished and diamond seeded titanium alloy substrates (Ti-6Al-4V). The experiments were repeated in duplicates to confirm reproducibility. The microwave power was 1.2 kW and the chamber pressure was 38 Torr for all depositions. This produces a sample deposition temperature of 700–800 °C. The deposition time for all samples was 4 h, and the film thickness was measured by in-situ optical interferometric technique [15]. The thickness of the samples was determined to be 2.2 μm for MCD, 3 μm for NSD, 1.2 μm for BDD, and 0.825 μm for BND.

For clarity the sample naming convention reflects the gas chemistry of each sample. Four types of diamond were grown in this study. Microcrystalline diamond (MCD) was grown with 500 SCCM of H_2_ and 88 SCCM of CH_4_. Nanostructured diamond (NSD) was grown with 500 SCCM of H_2_, 88 SCCM of CH, and 8.8 SCCM of N_2_. Diamond with boron and nitrogen additions (BND) was grown with 500 SCCM of H_2_, 88 SCCM of CH, 8.8 SCCM of N_2_, and 1 SCCM of diluted diborane mixture (10% B_2_H_6_ in H_2_). Diamond with only boron addition (BDD) was grown with 500 SCCM of H_2_, 88 SCCM of CH, and 1 SCCM of diluted diborane mixture (10% B_2_H_6_ in H_2_). The boron chemistry provides a gas composition with approximately 170 ppm of diborane. When nitrogen is used it comprises approximately 14,700 ppm of the gas.

Optical Emission Spectroscopy was performed on the various plasma conditions to obtain information about the active gas species in the plasma. This was performed with an Acton Research SpectaPro 500i spectrograph (Princeton Instruments, Trenton, NJ, USA) with a 1200 gr/mm grating blazed at 300 nm and entrance slit set at 10 microns.

The XRD data was collected using a Philips X-Pert MPD machine (Panalytical, Westborough, MA, USA) with a Cu anode (λ = 1.5418 Å), Ni filter, and a graphite monochromator. The diffractometer was set up with an *x*-ray incident angle of 3° (fixed) and then the diffracted beam was scanned over 33°–78°. This produces an increased signal from the thin-film and decreased signal from the bulk alloy. The FWHM of the diamond (111) peak at 44° 2θ was used for qualitative observation of the crystal size. FWHM analysis of the D(111) peaks was performed using Matlab (MATLAB^®^ and Statistics Toolbox Release 2012b, The MathWorks, Inc., Natick, MA, USA) by isolating the 402 data points between 43 ° 2θ and 45 ° 2θ and fitting an appropriate Lorentzian curve to each of the data sets [16]. Then, for each computed Lorentzian curve, the locations of the two points (in ° 2θ) at half of the peak’s maximum value were computed. The difference between these two values was taken as the highest-precision FWHM value possible.

SEM imaging was performed on the samples to examine the surface morphology and obtain a grain size measurement. The instrument used was a FEI QuantaTM 650 FEG (Thermo Fisher Scientific, Hillsboro, OR, USA) and all images were taken at 20 kV beam voltage.

## 5. Conclusions

We have carried out microwave plasma chemical vapor deposition of diamond films on Ti-6Al-4V alloy substrates using H_2_/CH_4_/N_2_/B_2_H_6_ precursor gases. A standard nanostructured diamond film was grown using H_2_/CH_4_/N_2_ precursor gases while the addition of B_2_H_6_ causes a morphological transition with crystal grain size changing from nanometer to micron scale. This abrupt change in morphology happens with addition of only 170 ppm level of B_2_H_6_ in the plasma. A detailed study of various plasma species by optical emission spectroscopy has correlated a reduction in CN species to this morphological transition of crystal grain size from nanometer to micron scale. Our study has clearly identified the role of CN species in the synthesis of nanostructured diamond and suppression of CN species by addition of boron to the plasma. This drop in CN is attributed to the increase of BN radicals in the plasma which absorb the nitrogen in the plasma. The present study also offers a new control of diamond film morphology and electrical properties for various applications. 

## Figures and Tables

**Table 1:** Measured values for FWHM (Full Width at Half Maximum) of diamond (111) Bragg *x*-ray peak and lattice parameter “*a*” in Angstrom for cubic diamond.

Sample	MCD	NSD	BND	BDD
FWHM (° 2θ)	0.305	0.9275	0.295	0.332
*a* (Å)	3.5708	3.5723	3.5782	3.5771

## References

[1] Samudrala G.K., Vohra Y.K., Walock M.J., Miles R. (2014). Rapid Growth of Nanostructured Diamond Film on Silicon and Ti-6Al-4V Alloy Substrates. Materials.

[2] Liang Q., Harrison J.G., Vohra Y.K. (2008). Modeling of Nitrogen/diborane/methane/hydrogen Plasma for Nanocrystalline Diamond Growth: Comparison with Experimental Data. Diam. Relat. Mater..

[3] Collins A.T., Williams A.W.S. (1971). The nature of the acceptor centre in semiconducting diamond. J. Phys. C: Solid State Phys..

[4] Ullah M., Ahmed E. (2012). Influence of boron carbide on properties of CVD-diamond thin films at various deposition pressures. Curr. Appl. Phys..

[5] Klein T., Achatz P., Kacmarcik J., Marcenat C., Gustafsson F., Marcus J., Bustarret E., Pernot J., Omnes F., Sernelius B.E. (2007). Metal-insulator transition and superconductivity in boron-doped diamond. Phys. Rev. B.

[6] Ekimov E.A., Sidorov V.A., Bauer E.D., Mel’nik N.N., Curro N.J., Thompson J.D., Stishov S.M. (2004). Superconductivity in diamond. Nature.

[7] Ashcheulov P., Sebera J., Kovalenko A., Petrak V., Fendrych F., Nesladek M., Taylor A., Vlckova Zivcova Z., Frank O. (2013). Conductivity of boron-doped polycrystalline diamond films: Influence of specific boron defects. Eur. Phys. J. B.

[8] Barnard A.S., Sternberg M. (2006). Substitutional boron in nanodiamond, bucky-diamond, and nanocrystalline diamond grain boundaries. J. Phys. Chem. B.

[9] Bhattacharyya S., Auciello O., Birrell J., Carlisle J.A., Curtiss L.A., AGoyette A.N., Gruen D.M., Krauss A.R., Schlueter J., Sumant A. (2001). Synthesis and characterization of highly-conducting nitrogen-doped ultrananocrystalline diamond films. Appl. Phys. Lett..

[10] Birrell J., Gerbi J.E., Auciello O., Gibson J.M., Gruen D.M., Carlisle J.A. (2003). Bonding structure in nitrogen doped ultrananocrystalline diamond. J. Appl. Phys..

[11] Catledge S.A., Vohra Y.K. (1999). Effect of nitrogen addition on the microstructure and mechanical properties of diamond films grown using high-methane concentrations. J. Appl. Phys..

[12] Brunet F., Germi P., Pernet M., Deneuville A., Gheeraert E., Laugier F., Burdin M., Rolland G. (1998). The effect of boron doping on the lattice parameter of homoepitaxial diamond films. Diam. Relat. Mater..

[13] Sakaguchi I., Nishitani-Gamo M., Loh K.P., Yamamoto K., Haneda H., Ando T. (1998). Effect of oxygen addition on boron incorporation on semiconductive diamond CVD. Diam. Relat. Mater..

[14] Bohr S., Haubner R., Lux B. (1996). Influence of nitrogen additions on hot-filament chemical vapor deposition of diamond. Appl. Phys. Lett..

[15] Catledge S.A., Comer W., Vohra Y.K. (1998). In situ diagnostics of film thickness and surface roughness of diamond films on a Ti–6Al–4V alloy by optical pyrometry. Appl. Phys. Lett..

[16] Mathworks Lorentzfit(x-y-varargin). https://www.mathworks.com/matlabcentral/fileexchange/33775-lorentzfit-x-y-varargin.

